# Genetic and Molecular Basis of Individual Differences in Human Umami Taste Perception

**DOI:** 10.1371/journal.pone.0006717

**Published:** 2009-08-21

**Authors:** Noriatsu Shigemura, Shinya Shirosaki, Keisuke Sanematsu, Ryusuke Yoshida, Yuzo Ninomiya

**Affiliations:** Section of Oral Neuroscience, Graduate School of Dental Science, Kyushu University, Fukuoka, Japan; Duke Unviersity, United States of America

## Abstract

Umami taste (corresponds to savory in English) is elicited by L-glutamate, typically as its Na salt (monosodium glutamate: MSG), and is one of five basic taste qualities that plays a key role in intake of amino acids. A particular property of umami is the synergistic potentiation of glutamate by purine nucleotide monophosphates (IMP, GMP). A heterodimer of a G protein coupled receptor, TAS1R1 and TAS1R3, is proposed to function as its receptor. However, little is known about genetic variation of *TAS1R1* and *TAS1R3* and its potential links with individual differences in umami sensitivity. Here we investigated the association between recognition thresholds for umami substances and genetic variations in human *TAS1R1* and *TAS1R3*, and the functions of TAS1R1/TAS1R3 variants using a heterologous expression system. Our study demonstrated that the TAS1R1-372T creates a more sensitive umami receptor than -372A, while TAS1R3-757C creates a less sensitive one than -757R for MSG and MSG plus IMP, and showed a strong correlation between the recognition thresholds and in vitro dose - response relationships. These results in human studies support the propositions that a TAS1R1/TAS1R3 heterodimer acts as an umami receptor, and that genetic variation in this heterodimer directly affects umami taste sensitivity.

## Introduction

Taste is the sensory system devoted to the evaluation of the quality of potential foods and to the determination of whether they should be ingested or rejected. The sense of taste is widely believed to be composed of a small number of primary qualities, in particular sweet, sour, bitter, salty and umami (a Japanese word that roughly translates into delicious and corresponds in many ways to savory in English). Of these five taste qualities or modalities, umami is elicited by L-glutamate, typically as its Na salt (monosodium glutamate: MSG), some amino acids and purine nucleotides (such as IMP and GMP). A salient feature of umami taste in rodents and humans is their impressive potentiation by purine nucleotides [1], [2]. Many protein (amino acid) rich foods, including meat, milk and seafood taste delicious (umami) to humans, and are attractive to rodents and other animals, suggesting that umami perception plays a particularly key role in ingestion of amino acids (especially L-glutamate) which act as biosynthetic precursors of various molecules, metabolic fuels and neurotransmitters.

Taste sensitivity to umami substances varies among individual humans. Distribution of individual MSG thresholds shows a bi-modal curve, and taste thresholds of MSG differ about 5-fold between taster and hypotaster groups [3]. However, the cause of individual differences in umami perception is unknown.

TAS1R1 (taste receptor type1 member 1, encoded by *TAS1R1*) and TAS1R3 (taste receptor type1 member 3, encoded by *TAS1R3*) are G protein-coupled receptors (GPCR) and belong to family C [4]–[9], which includes the metabotropic glutamate receptors (mGluRs), the GABA_B_ receptor, the calcium-sensing receptor, and some pheromone receptors [10]. A heterodimer of TAS1R1 and TAS1R3 (TAS1R1/TAS1R3) functions as an umami taste receptor in humans and rodents [11]–[15].

Here we provide additional evidence favoring TAS1R1/TAS1R3 as an umami receptor and to elucidate underlying molecular mechanisms for individual variation in umami sensitivity, by investigating potential associations between the taste recognition thresholds for umami substances and genetic variations of *TAS1R1* and *TAS1R3*, and functionality of TAS1R1/TAS1R3 variants using heterologous expression system.

## Results

### Taste recognition thresholds for umami compounds

**Figure 1:**
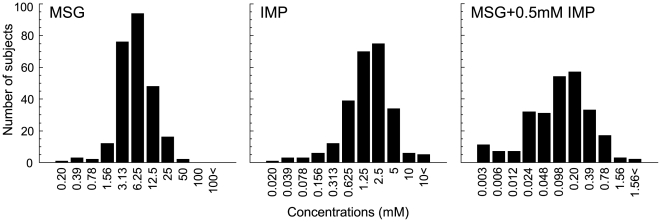
Distributions of individual MSG, IMP and MSG+0.5 mM IMP taste recognition thresholds (254 Japanese subjects). Bin width concentrations correspond to 0.3 log units. The subjects exhibiting recognition threshold over 100 mM MSG, 10 mM IMP, 1.56 mM MSG+0.5 mM IMP are presented as 100<, 10<and 1.56<, respectively.

Distributions of MSG, IMP and MSG plus 0.5 mM IMP (M+I) taste recognition thresholds are presented in Figure 1. The means (±S.E.) recognition thresholds for MSG, IMP and M+I were 7.66±0.43, 2.15±0.12 and 0.20±0.016, respectively. No significant differences in the mean thresholds between males and females were found (MSG: male vs. female = 8.27±0.60 vs. 6.91±0.62, *p* = 0.12; IMP: 2.17±0.17 vs. 2.11±0.17, *p* = 0.80; M+I: 0.20±0.02 vs. 0.19±0.02, *p* = 0.81, *t*-test). Therefore, data for both genders were combined. A normal distribution was found for both MSG and IMP, whereas a bi-modally like distribution with the node between 0.048 and 0.098 mM, was observed for M+I.

### Genotype and allele frequencies of SNPs in human TAS1R1 and TAS1R3

The entire coding regions of human *TAS1R1* (2526 bps) and *TAS1R3* (2559 bps) sequences were examined. The results are presented in Table 1. Comparisons of aligned exonic sequences revealed 7 SNPs in *TAS1R1* (2 synonymous and 5 non-synonymous) and 10 SNPs in *TAS1R3* (6 synonymous and 4 non-synonymous). No significant deviation from Hardy-Weinberg Equilibrium (HWE) was observed in all SNPs. Of these, 6 SNPs (amino acid position 191, 347 and 372 in TAS1R1, 416, 757 and 813 in TAS1R3) have been reported in the dbSNP of NCBI (http://www.ncbi.nlm.nih.gov/SNP/), 7 SNPs (191, 347 and 372 in TAS1R1, 416, 573, 659 and 757 in TAS1R3) had been reported by Kim *et al.* [16], while 9 SNPs (12, 85, 139 and 173 in TAS1R1, 74, 110, 551, 690 and 751 in TAS1R3) were newly identified in this study. Out of 9 SNPs with an amino acid substitution, 6 SNPs showed minor allele frequencies of less than 1.5%, while 3 SNPs (12 and 372 in TAS1R1, 757 in TAS1R3) showed minor allele frequencies of over 5.0% (6.3, 39.2 and 9.0%, respectively). The minor allele frequencies of SNPs at 372 in TAS1R1 and 757 in TAS1R3 were almost the same as those shown in dbSNP database [35.6% (ss68757616) and 8.0% (ss1867265), respectively]. Thus, 3 SNPs (Q12H, A372T in TAS1R1 and R757C in TAS1R3) were considered as common SNPs among the sample we studied and were selected for further association and functional analyses.

**Table 1:** Genotype and allele frequencies of SNPs in human taste receptors, *TAS1R1* and *TAS1R3*.

Genes	Position	Nucle-	Amino	Genotype			HWE	Allele	
	mRNA	Amino	dbSNP	otide	acid	frequency		frequency (%)
		acid	rs#		encoded						
*TAS1R1*											
NM_138697	36	12		G	Gln	G/G	G/C	C/C		G	C
				C	His	236	32	1	0.94	93.7	6.3
	255	85		G	Glu	G/G	G/A	A/A		G	A
				A	Glu	100	1	0	0.96	99.5	0.5
	416	139		C	Thr	C/C	C/T	T/T		C	T
				T	Met	98	3	0	0.88	98.5	1.5
	519	173		C	Ser	C/C	C/T	T/T		C	T
				T	Ser	104	1	0	0.96	99.5	0.5
	572	191	rs61744700	A	Asn	A/A	A/G	G/G		A	G
				G	Ser	104	1	0	0.96	99.5	0.5
	1039	347	rs10864628	G	Glu	G/G	G/A	A/A		G	A
				A	Lys	119	1	0	0.96	99.6	0.4
	1114	372	rs34160967	G	Ala	G/G	G/A	A/A		G	A
				A	Thr	96	106	43	0.15	60.8	39.2
*TAS1R3*											
NM_152228	220	74		C	Leu	C/C	C/T	T/T		C	T
				T	Leu	166	16	1	0.38	95.1	4.9
	329	110		T	Met	T/T	T/C	C/C		T	C
				C	Thr	187	1	0	0.97	99.7	0.3
	1248	416	rs3813210	C	Pro	C/C	C/T	T/T		C	T
				T	Pro	54	7	0	0.63	94.3	5.7
	1652	551		G	Ser	G/G	G/A	A/A		G	A
				A	Asn	51	1	0	0.94	99.0	1.0
	1719	573		C	Leu	C/C	C/T	T/T		C	T
				T	Leu	55	5	0	0.74	95.8	4.2
	1977	659		C	Phe	C/C	C/T	T/T		C	T
				T	Phe	54	1	0	0.95	99.0	1.0
	2070	690		G	Leu	G/G	G/A	A/A		G	A
				A	Leu	103	1	0	0.96	99.5	0.5
	2253	751		G	Val	G/G	G/A	A/A		G	A
				A	Val	113	1	0	0.96	99.6	0.4
	2269	757	rs307377	C	Arg	C/C	C/T	T/T		C	T
				T	Cys	205	36	4	0.11	91.0	9.0
	2438	813	rs34810828	G	Arg	G/G	G/A	A/A		G	A
				A	Lys	103	1	0	0.96	99.5	0.5

### Association between three SNPs of human TAS1Rs and taste recognition thresholds for umami compounds

Based on statistical criteria we established for taste recognition threshold measurements for umami compounds [higher or lower than the mean±half a standard deviation for MSG and IMP, the node between 0.048 and 0.098 mM for M+I], samples examined both thresholds and a genotype for amino acid position 12 and 372 in TAS1R1 or 757 in TAS1R3 were divided into two groups [MSG: low taste recognition threshold group (0.2∼3.13 mM, n = 86∼91) vs. high (12.5∼50 mM, n = 63∼67), IMP: low (0.02∼0.625 mM, n = 57∼62) vs. high (5.0∼40 mM, n = 43∼45), M+I: low (0.003∼0.048 mM, n = 80∼85) vs. high (0.098∼12.5 mM, n = 151∼160)] and analyzed statistically (Table 2). Significant associations were observed between the genotype at 372 in TAS1R1 and recognition thresholds for MSG (χ^2^ = 14.6, *p* = 0.0007) and M+I (χ^2^ = 6.19, *p* = 0.04), and between allele frequency at 757 in TAS1R3 and recognition threshold for IMP (χ^2^ = 6.19, *p* = 0.01). Regarding the genotype and allele frequency at 12 in TAS1R1, no significant differences between low and high taste recognition threshold groups were observed for MSG, IMP or M+I (χ^2^ = 0.0005∼0.12, *p* = 0.83∼0.98).

**Table 2:** Effect of SNPs in human *TAS1R1* and *TAS1R3* on phenotype.

Genes	Amino	Threshold	(mM)		Genotype					Allele			
	acid				frequency			χ^2^	*p*	frequency		χ^2^	*p*
*TAS1R1*	12	MSG			G/G	G/C	C/C			G	C		
			∼3.125		76	11	0	0.04	0.98	163	11	0.009	0.93
			12.5∼		59	7	1			125	9		
		IMP			G/G	G/C	C/C			G	C		
			∼0.625		54	8	0	0.12	0.94	116	8	0.05	0.83
			5.0∼		40	4	1			84	6		
		MSG+0.5 IMP			G/G	G/C	C/C			G	C		
				∼0.05	74	10	0	0.12	0.94	158	10	0.0005	0.98
				0.098∼	143	17	1			303	19		
	372	MSG			G/G	G/A	A/A			G	A		
				∼3.125	43	26	22	14.6	0.0007*	112	70	0.19	0.66
				12.5∼	19	40	7			78	54		
		IMP			G/G	G/A	A/A			G	A		
				∼0.625	26	23	12	2.38	0.3	75	47	0.23	0.63
				5.0∼	13	24	6			50	36		
		MSG+0.5 IMP			G/G	G/A	A/A			G	A		
				∼0.05	34	29	22	6.49	0.04*	97	73	1.72	0.19
				0.098∼	64	74	22			202	118		
*TAS1R3*	757	MSG			C/C	C/T	T/T			C	T		
				∼3.125	78	6	2	3.94	0.14	162	10	2.76	0.09
				12.5∼	50	12	1			112	14		
		IMP			C/C	C/T	T/T			C	T		
				∼0.625	52	4	1	5.38	0.07	108	6	6.19	0.01*
				5.0∼	31	11	2			73	15		
		MSG+0.5 IMP			C/C	C/T	T/T			C	T		
				∼0.05	70	8	2	2.74	0.25	148	12	0.91	0.34
				0.098∼	122	29	1			273	31		

### Distribution of taste recognition thresholds for umami substances in groups defined by human TAS1R1/TAS1R3 haplotypes

**Figure 2:**
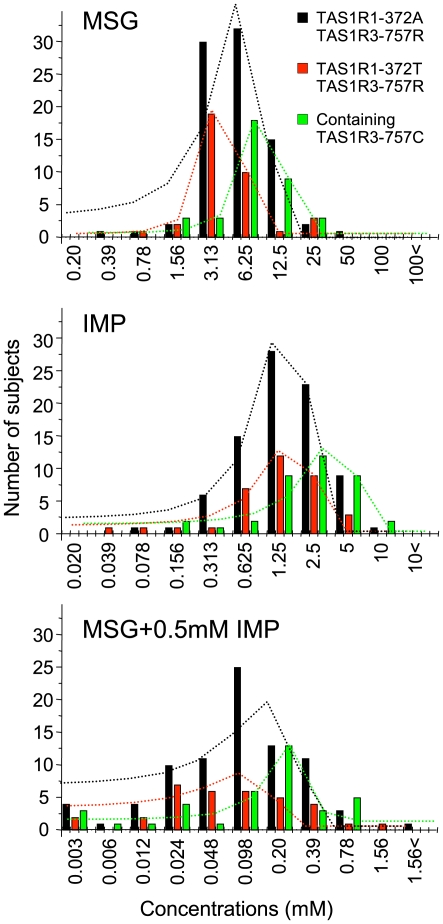
Distributions of individual MSG, IMP and MSG+0.5 mM IMP taste recognition thresholds for three groups defined by TAS1R1-372 and TAS1R3-757 haplotypes. Black, red and green bars indicate human TAS1R1-372A/TAS1R3-757R homozygotes (n = 84), TAS1R1-372T/TAS1R3-757R homozygotes (n = 37) and homo- and heterozygotes containing the TAS1R3-757C (n = 39), respectively. Black, red and green dotted lines indicate the distribution curves for TAS1R1-372A/TAS1R3-757R homozygotes, TAS1R1-372T/TAS1R3-757R homozygotes and homo- and heterozygotes containing the TAS1R3-757C, respectively, which were obtained by gaussian fit analysis. Bin width concentrations correspond to 0.3 log units. The subjects exhibiting recognition threshold over 100 mM MSG, 10 mM IMP, 1.56 mM MSG+0.5 mM IMP are presented as 100<, 10<and 1.56<, respectively.

Next, to analyze effects of amino acid substitutions (TAS1R1-A372T, TAS1R3-R757C) associated with differences in umami taste recognition thresholds, subjects were divided into three groups according to their TAS1R1-372/TAS1R3-757 haplotypes [(1) AR homozygotes (372A/757R, n = 84), (2) TR homozygotes (372T/757R, n = 37) and (3) TAS1R3-757C [homo- and heterozygotes containing TAS1R3-757C, n = 39. Because the numbers of AC and TC homozygotes were small (n = 0 and 1, respectively), heterozygotes containing TAS1R3-757C {AC/TC (n = 2), AR/AC (n = 14), TR/TC (n = 5) and AR/TC (n = 17)} were added to AC and TC homozygotes], and distributed (Figure 2). ANOVAs showed significant differences in the mean values (±S.E.) among AR, TR and TAS1R3-757C in MSG [TR (4.65±0.47 mM), AR (6.23±0.45) and TAS1R3-757C (8.43±0.98); F (2, 156) = 7.0, *p*<0.01], IMP [TR (1.60±0.21), AR (1.78±0.15) and TAS1R3-757C (2.92±0.39); F (2, 156) = 7.8, *p*<0.001] and M+I [TR (0.12±0.03), AR (0.18±0.02) and 757C (0.23±0.04); F (2, 155) = 3.2, *p*<0.05]. Post hoc Student's *t*-test indicated significant differences; MSG: AR vs. TR (*p* = 0.017), AR vs. 757C (*p* = 0.001), TR vs. 757C (*p* = 0.045), IMP: AR vs. 757C (*p* = 0.004), TR vs. 757C (*p* = 0.008), M+I: AR vs. 757C (*p* = 0.026). Gaussian fit analysis also demonstrated that the mean values of recognition thresholds were different among three groups [MSG: TR (4.38±0.29 mM)<AR (7.51±0.44)<TAS1R3-757C (8.60±0.20), M+I: TR (0.069±0.01)<AR (0.13±0.009)<TAS1R3-757C (0.22±0.026). For IMP, the mean value of the recognition thresholds of TR was almost the same as AR (1.73±0.13 and 1.77±0.06, respectively), while that of TAS1R3-757C (3.40±0.19) was higher than those of TR and AR. These results suggest that the *TAS1R1*-372T allele may be a more sensitive allele than the -372A allele for MSG and M+I, but not for IMP. The *TAS1R3-*757C allele may be the less sensitive allele than the *-*757R allele for MSG, IMP and M+I. For the other basic taste stimuli (sucrose, NaCl, HCl and PTC), no such differences in distributions were observed among TR, AR and TAS1R3-757C [see supporting information (SI) Figure S1].

### Functional expression of human TAS1R1 and TAS1R3

In order to assess the effects of two amino acid mutations at the molecular level, we used a functional expression analysis using human embryonic kidney 293 (HEK) cells as has been used in the characterization of bitter, sweet and umami taste receptors [11], [12], [15], [17]. Major type, *TAS1R1*-372A and *TAS1R3*-757R, were transiently transfected in HEK cells along with G α16-i3. We then monitored activation (Ca^2+^ mobilization) by a Ca^2+^ indicator dye, using various tastants (Figure S2 *A*). MSG (5∼100 mM) elicited transient intracellular calcium increases in the transfected cells in a concentration dependent manner. Synergism between MSG and IMP is a hallmark of umami taste [1], [2]. IMP (1, 10 mM) alone did not activate TAS1R1/TAS1R3 (data not shown), but 0.5 mM IMP potentiated the TAS1R1/TAS1R3 response to 1 and 20 mM MSG (Figure S2 *A* and *B*). The transfected HEK cells did not respond to 50 mM sucrose, 50 mM NaCl (Figure S2 *A*), 50 mM glucose, 1 mM saccharin and 0.3 mM SC45647 (data not shown). Mock-transfected HEK cells (without the three genes, G α16-i3 alone, G α16-i3/*TAS1R1* without *TAS1R3*, G α16-i3/*TAS1R3* without *TAS1R1*) did not respond to MSG and M+I (Figure S2 *B* and *C*). Lactisole is a sweet and umami taste inhibitor [15], [18]. The inhibition effect of lactisole was clearly observed in the transfected cells (Figure S5).

### Functional characterization of amino acid substitutions, A372T in Human TAS1R1 and R757C in TAS1R3

**Figure 3:**
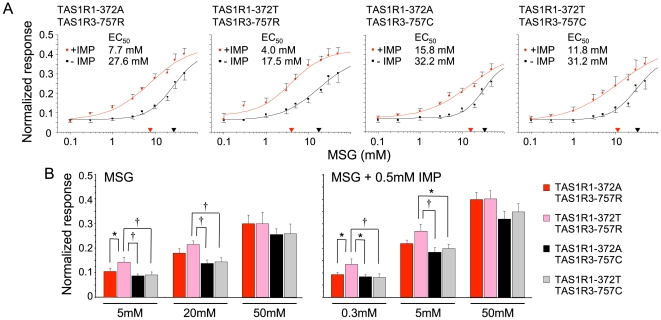
Concentration-response relationships of the calcium concentrations in HEK293 cells transfected with human *TAS1R1* and *TAS1R3* variants after stimulation with increasing MSG, and MSG+0.5 mM IMP. (A) Dose-response curves of MSG, and MSG+0.5 mM IMP concentration series in cells expressing the TAS1R1/TAS1R3 variants [TAS1R1-372A, -372T, TAS1R3-757R and -757C]. Black and red lines indicate the responses to MSG and MSG+0.5 mM IMP, respectively. Responses have been normalized to those of isoproterenol (ISO, 10 µM), which activate the endogenous βadrenergic receptor [12]. Each point represented the mean (±S.E.) from 10∼18 independent experiments. Half maximal responses (EC_50_) for TAS1R1/TAS1R3 variants were calculated from the dose-response curves. The X-axis triangles present the EC_50_ values for MSG (black) and MSG+0.5 mM IMP (red). (B) The responses of TAS1R1/TAS1R3 variants at concentrations of MSG (5, 20, 50 mM) and MSG (0.3, 5, 50 mM)+0.5 mM IMP. The values are means (±S.E.) from 14∼18 independent experiments. Asterisks indicate statistically significant differences (*: *p*<0.05, †: *p*<0.01, Fisher's PLSD as a Post-hoc test).

To further examine the significant association between two amino acid substitutions and taste recognition threshold for umami compounds, we constructed mutant receptors encoding TAS1R1-372A, -372T, TAS1R3-757R and -757C. These constructs were tested for their responses to MSG and M+I. The concentration-response functions of cells transfected with different human TAS1R1/TAS1R3 variants are shown in Figure 3. We found that upon stimulation with MSG, cells containing TAS1R1/TAS1R3 variants responded with a robust transient increase of the intracellular-calcium levels. Synergisms between MSG and 0.5 mM IMP were observed in all cells with TAS1R1/TAS1R3 variants. To test whether there is functional difference in agonist sensitivity, we calculated the half maximal effective concentrations (EC_50_) (Figure 3*A*). Comparison of EC_50_ values demonstrated that: (1) 372T/757R (TR) showed about 1.5-fold more sensitive responses for MSG compared to the other variants (AR, AC and TC) (17.5 mM vs. 27.6∼32.2 mM), (2) receptor variants containing 372T showed about 1.5∼2-fold more sensitive responses for M+I compared to counter parts containing 372A (AR vs. TR or AC vs. TC), (3) receptor variants containing 757C showed about 2∼2.5-fold less sensitive responses for M+I compared to counter parts containing 757R (AR vs. AC or TR vs. TC).

We, next, statistically examined whether the amino acid substitutions affect the receptor function at low (3 mM MSG and 0.3 mM M+I, around the concentration of thresholds), middle (20 mM MSG and 5 mM M+I, around the concentration of EC_50_) and high concentrations (50 mM MSG and M+I, around the concentration eliciting maximal responses) (Figure 3*B*). ANOVA for the mean values showed significant differences among 4 receptor variants at 5, 20 mM MSG, and 0.3, 5 mM M+I [5 mM MSG: means±S.E.: 0.88±0.008∼0.145±0.018, F (3, 58) = 3.651, *p*<0.05; 20 mM MSG: 0.139±0.013∼0.216±0.014, F (3, 58) = 5.313, *p*<0.01; 0.3 mM M+I: 0.082±0.01∼0.135±0.022, F (3, 58) = 3.19, *p*<0.05; 5 mM M+I: 0.185±0.018∼0.271±0.027, F (3, 56) = 3.70, *p*<0.05], but not at 50 mM MSG and M+I [50 mM MSG: 0.258±0.022∼0.302±0.046, F (3, 59) = 0.45, *p* > 0.05; 50 mM M+I: 0.321±0.031∼0.403±0.032, F (3, 60) = 1.615, *p* > 0.05]. Post-hoc Fisher's PLSD test indicated significant differences; 5 mM MSG: AR vs. TR, TR vs. AC and TC; 20 mM MSG: TR vs. AC and TC; 0.3 mM M+I: AR vs. TR, TR vs. AC and TC; 5 mM M+I: TR vs. AC and TC (*p*<0.05). We also examined the effect of Q12H amino acid substitution of TAS1R1, which was expected to serve as a negative control, because we found no association between the genotypes (or allele frequencies) and taste recognition thresholds. The Q12H amino acid mutation of TAS1R1 did not affect MSG and M+I responses (Figure S3).

## Discussion

Distributions of MSG, IMP and M+I thresholds showed that individual subjects exhibited very different levels of sensitivity. Hence, large variations in recognition threshold sensitivity to umami substances were demonstrated. The distribution of MSG and M+I (Figure 1) may be made up of combinations of the varying underlying distributions of the TAS1R1-372 and TAS1R3-757 haplotypes, because we found only 2 SNPs (associated with differences in taste recognition thresholds) with high minor allele frequencies in human *TAS1R1* and *TAS1R3*. Distributions of the 3 groups divided according to TAS1R1-A372T and TAS1R3-R757C haplotypes showed 3 types of curves with about 2∼3-fold different mean values in MSG and M+I recognition thresholds (Figure 2). The mean values were TR<AR<TAS1R3-757C. Interestingly, the distribution of the mean values for IMP alone (TR = AR<TAS1R3-757C) was different compared with MSG (M+I). These results suggest that amino acid position 372 in TAS1R1 may modulate the binding domain for MSG but not IMP, and that the binding region for MSG may differ from that for IMP. These possibilities may be supported by previous studies using TAS1R2/TAS1R3 sweet receptor chimeras and mutants, which showed that there are at least three potential binding sites in this heterodimeric receptor [15], [18]–[20], and by a recent study using TAS1R1/TAS1R3 mutants [21]. Amino acid position 757 in TAS1R3 may be involved with broader functions (see below) because of effects on all umami compounds.

The relationship between threshold and suprathreshold taste perception is not clear [22]–[24]. In this study, therefore, we statistically examined whether the amino acid substitutions affect the receptor function at both low and middle concentrations in addition to comparing EC_50_ values. The functional analysis using HEK293 cells revealed that the human TAS1R1-372T is more sensitive than -372A, while TAS1R3-757C is less sensitive than -757R at both low and middle concentrations of MSG (5 and 20 mM) and M+I (0.3 and 5 mM). The amino acid substitution, TAS1R1*-*Q12H, did not affect on MSG and M+I responses. Thus, a strong correlation between the taste recognition thresholds and in vitro dose - response relationships was observed. The association between TAS1R3-R757C and the recognition threshold for IMP alone remains unclear, because transfected HEK cells showed no responses to IMP alone [11], [12], [15]. The apparent umami taste attributed to IMP alone *in vivo* may actually results from the interaction of IMP with a small concentration of glutamate present in saliva [25].

TAS1R1 and TAS1R3 are GPCR and belong to family C [4]–[10]. The family C GPCR has a large extracellular region that recognizes a specific ligand molecule [26], [27]. A conserved domain search in the NCBI database indicated that 372 in TAS1R1 is present in the predicted ligand binding domain, suggesting that TAS1R1-A372T amino acid substitution may lead to a conformational change of ligand binding site for glutamate thereby affecting umami taste sensitivity.

Ca^2+^ responses to MSG and M+I were smaller in the cells transfected with TAS1R3-757C than in those with -757R, even with more sensitive TAS1R1-372T. The protein expression rates were not different between 757R and 757C (50∼55%, Figure S4). These results suggest that the TAS1R3-R757C amino acid substitution may affect general functions of TAS1R1/TAS1R3 such as cell surface expression, dimerization of TAS1R1 and TAS1R3 or the dynamic equilibrium between the active and resting conformations modulated by the presence/absence of ligand as shown in mGluR1 [26], [27].

Population diversities of SNPs in *TAS1R1* and *TAS1R3* have been reported by Kim *et al.* [3]. Minor allele frequencies of the SNP at 372 in TAS1R1 vary among eight populations; 10% in Cameroonian, 0% in Amerindian (native Americans), 25% in North European, 35% in Japanese, 5% in Russian, 35% in Hungarian, 40% in Chinese and 6% in Pakistani, whereas those at 757 in TAS1R3 showed no obvious difference among populations. These results suggest that there may be differences in umami sensitivity related with *TAS1R1*-A372T among populations in the world.

We showed that the *TAS1R1/TAS1R3* genetic variation affects umami sensitivity. However, this is probably not the only factor involved. For example, three splicing variants of TAS1R1 were reported in humans (GenBank accession: NM_177539.1, NM_177540.1 and NM_177541.1). Splice variants of metabotropic glutamate receptors, mGluR1 and mGluR4, have also been proposed as taste receptors for glutamate [28], [29]. It is possible that differences in expression of three TAS1R1 variants or genetic variations of *mGluR1* and *mGluR4* may cause differences in umami sensitivity.

Our study demonstrated that the TAS1R1-372T creates a more sensitive umami receptor than -372A, while TAS1R3-757C creates a less sensitive one than -757R for MSG and MSG plus IMP, and showed a strong correlation between the recognition thresholds and in vitro dose - response relationships. These results in human studies support the propositions that a TAS1R1/TAS1R3 heterodimer acts as an umami receptor, and that genetic variation in this heterodimer directly affects umami taste sensitivity.

## Materials and Methods

### Subjects

A total of 254 healthy, non-obese, non-diabetic Japanese volunteers [male/female: 141/113, age: 20–36, body mass index (BMI); 16.5–24.8] participated in this study. The purpose of the study as well as the methods and procedure were explained to the participants and the patients in this manuscript have given written informed consent. All protocols were approved by the local ethics committee at Kyushu University. Subjects were asked to refrain from consuming alcohol the day before testing, caffeine on the day of testing, and food during the experimentation period. The inclusion criteria were satisfactory state of oral hygiene, non-smoking, regular work, sleep, and meal schedules.

### Taste recognition threshold measurement

As MSG contains sodium, great care was taken to compare individual sensitivities to MSG relative to NaCl. Recognition thresholds were, therefore, measured for five qualities of taste using different concentrations of MSG (0.2∼400 mM), inosine 5′-monophosphate (IMP, 0.02∼40 mM), MSG in the presence of 0.5 mM IMP (M+I, 0.003∼12.5 mM), sucrose (0.2∼400 mM), NaCl (0.2∼400 mM), HCl (0.02∼40 mM) and PTC (0.001∼2.0 mM). The differences in concentrations in the present study were in 0.3 log steps, which mean step-wise increases in concentration by a factor of 2.0. All solutions were made up in distilled water and used at room temperature. The testing procedure was the staircase-method modified from Pasquet *et al.* [30]. Each subject evaluated all 7 tastants. The seven series of solutions were presented one after another within a single section. Within each series, the solutions with each 2.0 ml were applied to the whole tongue of each subject with a pipet in order of ascending concentrations. The subjects were asked not to swallow test solutions and rinse their mouth between two solutions with distilled water. The subject was requested to correctly name the taste quality in each series. Once the taste of two successive concentrations was recognized successfully, the subject was given the previous unrecognized concentration (first reversal). The points at which concentration sequence changed from a decrease to an increase or vice versa were designated “reversals”. The procedure was terminated after five reversals, and the threshold was calculated as the mean of the concentration values of the last four reversals.

### Genotyping

DNA samples were extracted from buccal cells by a genomic DNA purification kit (Gentra systems) after measurement of taste recognition thresholds. The entire coding regions of human *TAS1R1* and *TAS1R3* sequences were amplified with specific PCR primers (Table S1). Amplified PCR products were sequenced with 10 pmoles forward and reverse primers. Sequence analysis and contig assembly were performed with AssemblyLIGN (v.1.09, Oxford Molecular Group). Sequenced numbers of each region were at least fifty. Resulting data has been deposited in GenBank.

### Preparation of chimeras and point mutations

Intronless human *TAS1Rs* and Gαl6 expression constructs were generated in the pEF-DEST51 Gateway vector (Invitrogen) by genomic DNA-based methods. To subclone each gene into the vector, a Kozak cassette was introduced at the 5′ end before the start codon. Gαl6 chimeras were generated by PCR with mutagenic primers. The carboxyl-terminal tail of Gαl6, five-residue (EINLL) was replaced by its counterpart from Gαi3 (ECGLY; Gα16-i3), Gαgust (DCGLF; Gα16-gust) [12], and Gα16-gust44 was generated as described [31]. The integrity of all DNA constructs was confirmed by DNA sequencing. Point mutations in *TAS1Rs* were made using the same overlapping PCR strategy with mutagenic primers. In preliminary studies, the Gα16-i3 elicited larger and more reliable responses than did Gα16, Gα16-gust or Gα16-gust44 (data not shown), therefore, Gα16-i3 was used in all experiments.

### Functional expression

HEK293 cells were cultured at 37°C under a humidified atmosphere containing 5% CO_2_ in Dulbecco's modified Eagle's medium supplemented with 10% fetal bovine serum. To obtain reproducible Ca^2+^ responses, cells were split every 2 days before cells become confluent. Cells were discarded after 2 months of passages and new cells were prepared from frozen-stock. For calcium imaging experiments, cells were seeded onto a 35 mm recording chamber or poly-D-lysine coated 96-well black-wall, clear-bottom recording plates. After 24 hrs at 37°C, confluent cells (60∼70%) were washed in OptiMEM medium supplemented with GlutaMAX-I (invitrogen) and plasmid DNAs were transiently cotransfected into HEK293 cells using Lipofectamine2000 regent (invitrogen) (2.0 µl per 1.0 µg DNA). *TAS1Rs* (or their mutants) and Gαl6-i3 were transfected using 1.5 and 1.0 µg of plasmid for 35 mm recording chambers, 0.09 and 0.06 µg of plasmid per well for 96-well plates, respectively. The transfection efficiencies were estimated by visualizing DsRed fluorescence (data not shown) or by immunocytochemistry of TAS1R3, and were typically 50∼55% (Figure S4). Immuno staining and Ca^2+^ imaging assays were performed 36∼40 hrs after transfection.

### Ca^2+^ imaging

Two Ca^2+^ imaging systems were used. A bath perfusion system was used for kinetics of activation. Transfected cells in 35 mm recording chambers were washed in Hank's balanced salt solution (HBSS) containing 10 mM HEPES, pH 7.4, and loaded with 3.0 µM Fluo-4 acetoxymethyl ester (invitrogen) for 1.0 hr at 37°C. The dye-loaded cells were subjected to Ca^2+^ imaging. Taste solutions diluted in HBSS were applied sequentially to the cells for 30 s with a peristaltic pump at a flow rate of 1.5 ml/min, and fluorescence images were obtained using a S Fluor ×20/0.75 objective lens (Nikon) via a cooled-CCD camera (C6790, Hamamatsu Photonics) fitted to a TE300 microscope (Nikon). Acquisition and analysis of these fluorescence images used AquaCosmos software (v. 1.3, Hamamatsu Photonics). A 3 min interval was left between each tastant application to ensure that the cells were not desensitized as a result of the previous application of tastants. Responses were measured from 30 individual responding cells. Compounds were MSG (1∼100 mM), IMP (1, 10 mM), MSG (0.1∼50 mM) in the presence of 0.5 mM IMP, sucrose (Suc, 50 mM), glucose (50 mM), saccharin (1 mM), SC45647 (0.3 mM) and NaCl (50 mM). Isoproterenol (ISO, 10 µM) was used as positive control, which stimulates endogenous β-adrenergic receptors, providing that the Gα16-dependent signal transduction cascade was functional [12].

Another imaging system, FlexStation II (Molecular Devices) was used for TAS1R mutant analysis. Transfected cells in 96-well plates were washed once with HBSS and then loaded with 50 µl of FLIPR Calcium 4 (Molecular Devices) in HBSS with 20 mM HEPES (pH 7.4) for 1 hr at 37°C as described in the Kit manual. The dye-loaded cells were subjected to FlexStation II. Fluorescence changes (excitation at 485 nm, emission at 525 nm, and cutoff at 515 nm) were monitored at 2 sec intervals; 50 µl of HBSS supplemented with 2×tastants were added at 30 sec, and scanning was continued for an additional 120 sec. Fluorescence responses from 20∼30 sec after tastant addition were measured, corrected for background fluorescence measured before compound addition, normalized to the response to 10 µM ISO. Stimuli were tested at concentrations that do not elicit calcium responses from mock transfected Gα16 cells. Compounds were MSG (0.1, 0.3, 1, 3, 5, 10, 20, 30, 50 mM), MSG (0.1, 0.3, 1, 3, 5, 10, 20, 30, 50 mM) in the presence of 0.5 mM IMP. Lactisole (5 mM) was used as inhibitor of human TAS1R1/TAS1R3 [15], [18].

### Immunocytochemistry

HEK293 cells transfected with *TAS1Rs* (or their mutants) and Gα16-i3 were rinsed with phosphate-buffered saline (PBS) and then fixed for 5 min with 4% paraformaldehyde. After three washes with PBS, cells were treated in blocking solution containing 0.5% blocking reagent (Roche) and 0.1% tween20, and then incubated with anti-TAS1R3 antibody (sc-22458, 1∶50 dilution, Santa Cruz) for overnight at 4°C. After three washes with PBS, alkaline phosphatase-conjugated anti-goat secondary antibody (Jackson ImmunoResearch) was added for 1 hr at room temperature. After three washes with PBS, cells were immersed in alkaline phosphatase buffer consisting of 100 mM Tris/HCl (pH 9.5), 100 mM NaCl, and 50 mM MgCl_2_ for 5 min and then the signals were developed using nitroblue-tetrazolium chloride and 5-bromo-4-chloro-3-indolylphosphate as chromogenic substrates. The reaction was stopped by rinsing in TE buffer. The signal specificities of TAS1R3 were tested by using mock-transfected HEK cells as a negative control.

### Data analysis

Student's *t*-test was used for detection of differences in the mean recognition thresholds between males and females. The χ^2^ test was used for checking Hardy-Weinberg Equilibrium (HWE) for the genotype frequencies in samples to control genotyping errors, for detection of differences in genotype and allele frequencies between high and low taste recognition threshold groups. Yates'χ^2^ test was used when the number was less than 10. The mean values (the center±S.E. of gaussian curve on X-axis) in the distribution of recognition thresholds defined by TAS1R1/TAS1R3 haplotypes (TAS1R1-372A/TAS1R3-757R homozygotes, TAS1R1-372T/TAS1R3-757R homozygotes, homo- and heterozygotes containing TAS1R3-757C) were calculated using gauss fit function of Origin 5.0 (OriginLab Corp). For Ca^2+^ imaging analysis, data were normalized to response to 10 µM ISO, and mean values (±S.E.) were calculated from 10∼18 independent analyses. EC_50_ values were calculated from individual cumulative concentration-response data using sigmoid fit functions of Origin 5.0. One factor ANOVAs were conducted for detection of differences in the distributions of recognition thresholds for umami substances among 3 groups defined by TAS1R1/TAS1R3 haplotypes, and in the responses to 5, 20 and 50 mM MSG or 0.3, 5 and 50 mM M+I among TAS1R1/TAS1R3 variants in Ca^2+^ imaging analysis. When the ANOVA was significant, post-hoc Student's *t*-test or Fisher's PLSD test was performed to compare individual means at each umami compound or concentration. Significance level was 0.05.

## Supporting Information

Table S1 Nucleotide sequences for the primers used in sequencing and genotyping.
(0.05 MB DOC)

Figure S1 Distributions of individual sweet: sucrose (Suc), salt: NaCl, sour: HCl and bitter: phenylthiocarbamide (PTC) taste recognition thresholds for three groups defined by TAS1R1-372 and TAS1R3-757 haplotypes. Black, red and green bar indicate human TAS1R1-372A/TAS1R3-757R homozygotes (n = 84), TAS1R1-372T/TAS1R3-757R homozygotes (n = 37) and homo- and heterozygotes containing TAS1R3-757C (n = 39), respectively. Black, red and green dotted lines indicate the distribution curves for TAS1R1-372A/TAS1R3-757R homozygotes, TAS1R1-372T/TAS1R3-757R homozygotes and homo- and heterozygotes containing the TAS1R3-757C, respectively, which were obtained by gaussian fit analysis. Bin width concentrations correspond to 0.3 log units. The subjects exhibiting recognition threshold over 100 mM MSG, 10 mM IMP, 1.56 mM MSG+0.5 mM IMP are presented as 100<, 10<and 1.56<, respectively.
(0.30 MB EPS)

Figure S2 Increases in the calcium concentrations in HEK293 cells transfected with Gα16-i3, human TAS1R1-372A and TAS1R3-757R after stimulation with various stimuli. (A) HEK293 cells expressing Gα16-i3, TAS1R1 and TAS1R3 responded to MSG and MSG+0.5 mM IMP, not sucrose (Suc) and NaCl. IMP (1, 10 mM) alone did not activate TAS1R1/TAS1R3 (data not shown), but 0.5 mM IMP potentiated the TAS1R1/TAS1R3 response to 1 mM MSG. 10 µM isoproterenol (ISO) was used as positive control. Horizontal bars above the traces indicate the duration of tastant pulses. (B) Increase of the calcium concentrations after stimulation with 20 mM MSG and potentiation of 0.5 mM IMP to 20 mM MSG was observed in HEK293 cells transfected with Gα16-i3/TAS1R1/TAS1R3, not in the cells in the absence of the three genes, TAS1R1 and TAS1R3, TAS1R3 or TAS1R1, respectively. (C) Dose-response curves of MSG, and MSG+0.5 mM IMP concentration series on cells in the absence of TAS1R1 or TAS1R3. No obvious increases of the calcium concentrations were observed in the cells. Responses have been normalized to those of ISO (10 µM). The values are means (±S.E.) (n = 3).
(0.53 MB EPS)

Figure S3 Concentration-response relationships of the calcium concentrations in HEK293 cells transfected with human TAS1R1-12Q-372A/TAS1R3 -757R or TAS1R1-12H-372A/TAS1R3 -757R after stimulation with increasing MSG, and MSG+0.5 mM IMP. (A) Dose-response curves of MSG, and MSG+0.5 mM IMP concentration series in cells expressing the TAS1R1/TAS1R3 variants. Black and red lines indicate the responses to MSG and MSG+0.5 mM IMP, respectively. Responses have been normalized to those of isoproterenol (10 µM). Each point represented the mean (±S.E.) from 5∼9 independent experiments. The X-axis triangles present the EC50 values for MSG (black) and MSG+0.5 mM IMP (red). No obvious differences in the EC50 values were observed between TAS1R1-12Q and -12H variants. (B) The responses of TAS1R1/TAS1R3 variants at concentrations of MSG (5, 20, 50 mM) and MSG (0.3, 5, 50 mM)+0.5 mM IMP. The values are means (±S.E.) from 6∼9 independent experiments. No significant differences were observed between TAS1R1-12Q and -12H variants (p > 0.05, ANOVA).
(0.27 MB EPS)

Figure S4 Immunocytochemistry for proteins of TAS1R3 variants transfected in HEK293 cells. TAS1R3 variants (TAS1R3-757R and -757C) showed no obvious differences in the expression rates (50∼55%). The signal specificities of TAS1R3 were tested by using mock-transfected HEK cells (without TAS1R3) as a negative control.
(1.09 MB EPS)

Figure S5 Inhibition effect of lactisole on the responses of TAS1R1/TAS1R3 variants at 20 mM MSG+0.5 mM IMP. Lactisole has been reported as a sweet and umami inhibitor [15], [18]. The inhibition effect of lactisole (5 mM) was clearly observed in the transfected cells. Responses have been normalized to those of isoproterenol (10 µM). The values are means (±S.E.) (n = 3).
(0.24 MB EPS)
