# Supplementary material for: Genetic and Molecular Basis of Individual Differences in Human Umami Taste Perception
Source: PLoS One. 2009 Aug 21;4(8):e6717. doi: 10.1371/journal.pone.0006717 (PMC2725291; doi:10.1371/journal.pone.0006717)
Supplement: Table S1 — Nucleotide sequences for the primers used in sequencing and genotyping. (0.05 MB DOC) [file pone.0006717.s001.doc]

| Table S1. Nucleotide sequences for the primers used in sequencing and genotyping. | | | | | | |
| --- | --- | --- | --- | --- | --- | --- |
|  | Genes | Accession No. | Name | Forward | Reverse |  |
|  |  |  |  |  |  |  |
|  | *TAS1R1* | NT_01937 | r1-Ex1 | TCACAGAGCGTGGACAGAAC | AGCACTTAGGTGGCAAGTGG | |
|  |  |  | r1-Ex2 | CTGGACCGATGACCTCAAAG | AAGAGCAGTGACCACCCAAG | |
|  |  |  | r1-Ex3a | CTTCGCAGGTGATTTGTCAG | GCTTCTTCAAACGCCTTCAG | |
|  |  |  | r1-Ex3b | TGCTTTCAAGGACATCATGC | TTTATCCCCCTTGGCATTTAG | |
|  |  |  | r1-Ex4 | CTCCTCTCAGGAGGTTGGTG | CTCGCCTACGGAGTGTCTTC | |
|  |  |  | r1-Ex5 | ACCACCACTCTACCCATCCTG | CCCAGTGGCTGATTCTCTTG | |
|  |  |  | r1-Ex6a | ATCTTGCAGGCCCCTATTTC | AGAGGAAGGCCAGTATGAAGC | |
|  |  |  | r1-Ex6b | CTAACTTGGCTGGTGGTGTG | AGGCTCTGGGTTCAGACTTG | |
|  | *TAS1R3* | NT_077913 | r3-Ex1 | CTGACGCGCACAAACTTTC | CAGAGCAGGCCGTTTGAG |  |
|  |  |  | r3-Ex2 | CATCTGCGGTTCTGTGTGG | AAGAAGGAGGGGAAGGTCTCC | |
|  |  |  | r3-Ex3a | TGTCAGGAGATGCCTCTTGG | GGTGCGTCTTCACGTACTGG | |
|  |  |  | r3-Ex3b | CACCAGGTGAACCAGAGCAG | CACGTGGAAGGTCAGGTT |  |
|  |  |  | r3-Ex4 | ACCACGCCTGAGCTGGAG | CCCACGGGAGACTGGAAC | |
|  |  |  | r3-Ex5 | TGGAGTACGACCTGAAGCTG | ACTCATCCTGGCCACAAAAG | |
|  |  |  | r3-Ex6a | GAGCCCACAGGGTACAAGAC | GAAAGTGCCCAGGAAGCAG | |
|  |  |  | r3-Ex6b | TCAGAACTGCCTCTGAGCTG | GAGACACGGGTCATTGCTG | |
|  |  |  |  |  |  |  |
